# Draft Genome Sequence of a Sporulating and Motile Strain of *Lachnotalea glycerini* Isolated from Water in Québec City, Canada

**DOI:** 10.1128/genomeA.01059-17

**Published:** 2017-10-19

**Authors:** Andrée F. Maheux, Dominique K. Boudreau, Ève Bérubé, Maurice Boissinot, Frédéric Raymond, Stéphanie Brodeur, Jacques Corbeil, Sandra Isabel, Rabeea F. Omar, Michel G. Bergeron

**Affiliations:** a Centre de Recherche en Infectiologie de l’Université Laval, Axe Maladies Infectieuses et Immunitaires, Centre de Recherche du CHU de Québec-Université Laval, Québec City, Canada; b Centre de Recherche en Données Massives, Université Laval, Québec City, Canada; c Département de Microbiologie, Infectiologie et d’Immunologie, Faculté de Médecine, Université Laval, Québec City, Canada; d Département de Médecine Moléculaire, Université Laval, Québec City, Canada

## Abstract

*Lachnotalea glycerini* CCRI-19302 belongs to the genus *Lachnotalea*. The strain was isolated from a water sample harvested in Québec City, Canada. The genome assembly comprised 4,694,231 bp, with 34.6% GC content. This is the first documentation to report the genome sequence of a sporulating and motile strain of *L. glycerini*.

## GENOME ANNOUNCEMENT

*Lachnotalea glycerini* CCRI-19302 (GenBank accession number MF574095) has been isolated from a water sample harvested in Québec City, Canada. Comparative 16S rRNA gene sequence analysis showed an identity of 99.8% with the strain DLD10^T^ sequence (accession number MF953294) (1). Based on this criterion, strains CCRI-19302 and DLD10^T^ belong to the same species (2). However, divergences in phenotypic characteristics were observed between these two strains. Mainly, cultures on sheep blood agar, under anaerobic conditions at 35°C for 72 h, revealed terminal deforming spores, motility, and β-hemolysis for CCRI-19302 but not for DLD10^T^.

Genomic DNA of strain CCRI-19302 was isolated by using a BioSprint 15 DNA blood kit (Qiagen) automated with a KingFisher mL instrument (Thermo Fisher Scientific). Whole-genome sequencing was performed on an Illumina HiSeq 2500 instrument using SBS version 4 to sequence a 126-bp paired-end library (Nextera XT [Illumina]). A total of 33,579,313 reads were assembled *de novo* in 124 contigs using the Ray software (version 2.3.0) (3). The total genome length is 4,694,231 bp (*N*_50_, 70,853 bp), with an average G+C content of 34.6% (4). The draft genome sequence was annotated using the NCBI GenBank annotation pipeline (version 4.2) and the Rapid Annotations using Subsystems Technology (RAST) annotation server (version 2.0) (5). A total of 4,215 features were identified, including 13 rRNAs and 53 tRNAs. Of the 4,087 putative protein-coding sequences, 1,141 were assigned as hypothetical proteins. The genome of CCRI-19302 contained homologs to genes known to be involved in sporulation (6), motility (7), and hemolysis (YqfA protein, hemolysin III family). PCR confirmed the presence of master sporulation regulator Spo0A and YqfA homologs in both strains.

### Accession number(s).

The whole-genome shotgun project of *Lachnotalea glycerini* CCRI-19302 has been deposited at DDBJ/ENA/GenBank under the accession number NOKA00000000. The version described in this paper is version NOKA01000000.
